# The Differential Antiviral Activities of Chicken Interferon α (ChIFN-α) and ChIFN-β are Related to Distinct Interferon-Stimulated Gene Expression

**DOI:** 10.1371/journal.pone.0059307

**Published:** 2013-03-19

**Authors:** Hongren Qu, Limin Yang, Shanshan Meng, Lei Xu, Yuhai Bi, Xiaojuan Jia, Jing Li, Lei Sun, Wenjun Liu

**Affiliations:** 1 Key Laboratory of Pathogenic Microbiology and Immunology, Institute of Microbiology, Chinese Academy of Sciences, Beijing, China; 2 Graduate University of Chinese Academy of Sciences, Beijing, China; 3 China-Japan Joint Laboratory of Molecular Immunology and Molecular Microbiology, Institute of Microbiology, Chinese Academy of Sciences, Beijing, China; University of Barcelona, Spain

## Abstract

Chicken interferon α (ChIFN-α) and ChIFN-β are type I IFNs that are important antiviral cytokines in the innate immune system. In the present study, we identified the virus-induced expression of ChIFN-α and ChIFN-β in chicken fibroblast DF-1 cells and systematically evaluated the antiviral activities of recombinant ChIFN-α and ChIFN-β by cytopathic-effect (CPE) inhibition assays. We found that ChIFN-α exhibited stronger antiviral activity than ChIFN-β in terms of inhibiting the replication of vesicular stomatitis virus, Newcastle disease virus and avian influenza virus, respectively. To elucidate the mechanism of differential antiviral activities between the two ChIFNs, we measured the relative mRNA levels of IFN-stimulated genes (ISGs) in IFN-treated DF-1 cells by real-time PCR. ChIFN-α displayed greater induction potency than ChIFN-β on several ISGs encoding antiviral proteins and MHC-I, whereas ChIFN-α was less potent than ChIFN-β for inducing ISGs involved in signaling pathways. In conclusion, ChIFN-α and ChIFN-β presented differential induction potency on various sets of ISGs, and the stronger antiviral activity of ChIFN-α is likely attributed to the greater expression levels of downstream antiviral ISGs.

## Introduction

Chicken type I interferon (IFN) was the first cytokine discovered that is induced by heat-inactivated influenza virus in the chorio-allantoic membranes of chicken embryos, was named IFN for its ability to directly interfere with influenza virus replication [1], and the IFN gene was subsequently cloned from a cDNA library from chicken embryonic cells [2], [3]. Both ChIFN-α and ChIFN-β are chicken Type I IFNs, and share analogous biological and biochemical features with mammalian IFN-α and IFN-β [3], [4].

Members of the type I IFN family play antiviral roles by binding to a common receptor consisting of two subunits (IFNAR1 and IFNAR2) that are located on the membrane surface of most cells [5], [6], [7]. Different type I IFNs display markedly distinct antiviral, antiproliferative and pro-apoptotic activities despite sharing the same receptors [8], [9], [10], [11], [12]. This led to the question of how IFN-α and IFN-β function differentially while binding to a common receptor, and several studies focus on the binding affinity of IFNs for their receptor and the cascading effect of downstream signaling pathways [13], [14], [15]. Type I IFNs can form a ternary complex with IFNAR1 and IFNAR2. Upon formation of the ternary complex, downstream signaling is initiated through the JAK-STAT pathway, thus inducing the transcription of IFN-stimulated genes (ISGs) [16]. ISGs are the primary effectors of IFN cellular responses and responsible for their antiviral, antiproliferative and immunomodulatory functions [15], [17]. Based on microarray data from human and murine cell cDNA samples, large numbers of ISGs have been identified and classified into several categories according to the biological functions of their products, including antiviral proteins such as double-stranded RNA-activated protein kinase (PKR), myxovirus resistance protein (Mx), the 2′–5′ oligoadenylate synthetases (2′,5′-OAS), IFN receptors (IFNAR1 and IFNAR2), inflammatory factors (IL-6 and IL-8), signal transduction factors (IFN regulatory factors 1-IRF1, myeloid differentiation primary-response gene 88 (MyD88), and STAT1) and the major histocompatibility complex (MHC) [17]. Additionally, type I IFNs produce a positive feedback effect on IFN production in an autocrine manner, which is mediated by IRFs and further amplifies downstream IFN expression [18], [19]. In sum, the cellular effects of type I IFNs are mediated by ISGs products together with IFNs themselves.

Abundant evidence demonstrates that ChIFN-α inhibits the replication of many epidemic avian viruses, such as avian influenza viruses (AIV), infectious bursal disease virus (IBDV), infectious bronchitis virus (IBV), Marek’s disease virus (MDV) and Newcastle disease virus (NDV) [20], [21], [22], [23], [24]. Our group reported that ChIFN-α is a potential preventive and therapeutic antiviral agent that inhibits H9N2 AIV replication and induces antiviral ISGs expression even by oral administration [24], implying the value of commercial application of ChIFN-α. Although less is known about ChIFN-β in comparison to ChIFN-α with regard to antiviral studies, it has been reported that the anti-vesicular stomatitis virus (VSV) activity of ChIFN-β is approximately 20-fold lower than that of ChIFN-α on chicken embryo fibroblast clone (CEC-32) cells [3]. Moreover, ChIFN-α has a stronger protective effect against PR8 than ChIFN-β in chicken macrophages [25]. Nevertheless, the systematic comparative analysis between ChIFN-α and ChIFN-β and the mechanism for their different effects on cells remain unclear. We speculate that the transcriptional levels of ISGs may be one of the indicators for understanding the divergent antiviral statuses triggered by ChIFN-α and ChIFN-β.

In the present study, recombinant ChIFN-α and ChIFN-β were cloned and expressed in *Escherichia coli* Strain BL21 (DE3) and purified by gel-filtration. We then compared the antiviral activities of the recombinant IFNs at different time points, and antiviral activities against VSV, NDV and AIV on DF-1 cells and anti-VSV activities on different cell lines. The relative mRNA levels of representative ISGs in ChIFN-treated DF-1 cells were also analyzed by real-time PCR to illustrate the mechanism of differential antiviral activity between these two ChIFNs.

## Results

### Sequences and Modeled Structures Alignment of ChIFN-α and ChIFN-β

**Figure 1:**
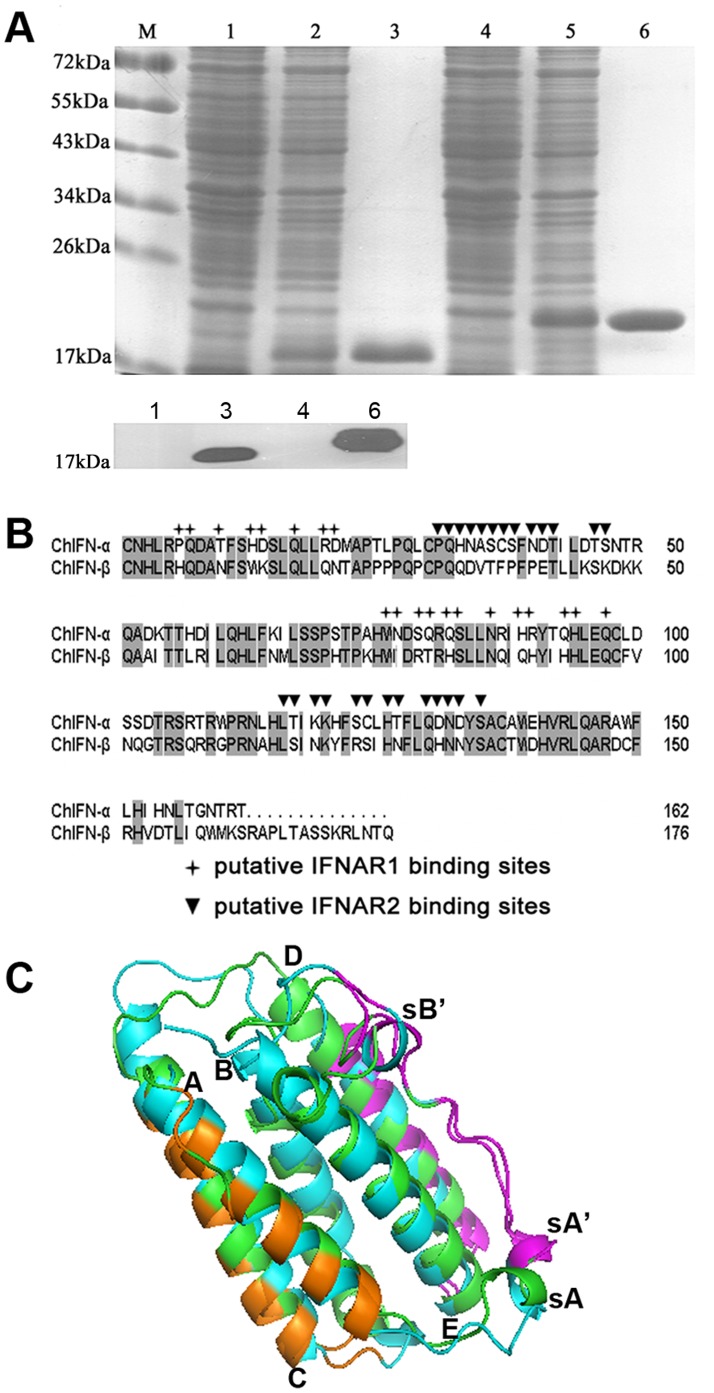
Sequences and structure alignment between ChIFN-α and ChIFN-β. (A) Expression and purification of ChIFN-α and ChIFN-β as identified by SDS-PAGE (top panel) and western blotting (bottom panel). Lane 1 and 4: total cell lysate from pre-induction samples of ChIFN-α and ChIFN-β, respectively. Lane 2 and 5: total cell lysate from induced samples of ChIFN-α and ChIFN-β, respectively. Lane 3 and 6: purified ChIFN-α and ChIFN-β, respectively, after gel filtration. The western blotting was performed using an anti-His monoclonal antibody. (B) The amino acid sequences of ChIFN-α and ChIFN-β were aligned by the program DNAMAN. They showed 50% identity, which was shaded gray. The putative IFNAR1 and IFNAR2 binding sites were marked with crosses and triangles, respectively. (C) The modeled structures were shown as cartoon using program PyMol and were aligned between ChIFN-α (green) and ChIFN-β (cyan). Both structures consist of five α-helices labeled A to E at the N-terminus of each helix. The small 3_10_ helix on the ChIFN-α AB loop was marked sA, and the 3_10_ helices in ChIFN-β were marked sA’ and sB’. The flexible N-terminus of ChIFN-α (C1 and N2) and C-terminus of ChIFN-β (R163-Q176) were removed from the structures. IFNAR1 binding sites marked in panel B were colored orange; IFNAR2 binding sites were colored magenta.

The ChIFN-α gene was amplified from chicken liver as previously described [24]. The ChIFN-β gene was amplified from the cDNA of VSV-infected DF-1 cells. Recombinant ChIFN-α and ChIFN-β, without signal peptides, were successfully expressed in *E. coli* and purified by gel filtration (Figure 1A, top panel); the purified proteins were identified by western blotting using an anti-His monoclonal antibody (Figure 1A, bottom panel). The deduced amino acid sequences of the ChIFNs were 100% identical with the deposited sequences in GenBank (GenBank-ID for ChIFN-α: ABB05335; and ChIFN-β: AAX83679.1). The two ChIFNs amino acid sequences were aligned by DNAMAN and displayed 50% identity. Their putative receptor binding sites were located according to the binding site descriptions for ChIFN-α and ChIFN-β from the NCBI protein database and sequence alignments between the ChIFNs and human IFN (the putative receptor interaction domains for human IFNα-2a have been found based on sequence alignments and site-directed mutagenesis [26]) (Figure 1B). Both ChIFN-α and ChIFN-β likely contain 20 residues that interact with IFNAR1 and 27 residues that interact with IFNAR2. Of these putative interacting amino acids, 13 (out of 20) involved in IFNAR1 binding and 17 (out of 27) involved in IFNAR2-binding are divergent between ChIFN-α and ChIFN-β, indicating a possible difference between the two ChIFNs in terms of their receptor binding affinities.

Despite limited sequence identities among human and chicken IFNs, the three-dimensional structures of ChIFN-α and ChIFN-β were successfully modeled on the basis of human IFN-α2 (PDB-ID: 2KZ1, chain A) and human IFN-β (PDB-ID:1AU1), respectively. The modeled structures of ChIFN-α and ChIFN-β were then superimposed in PyMOL (http://www.pymol.org/). As expected, many similarities were observed between the two ChIFNs in terms of their overall folds. Both proteins exhibit a typical helical structure, containing five α-helices (labeled A to E), exclusive of strand elements (Figure 1C). Helices A, B, C and E form a common four-α-helix bundle with an up-up-down-down topology and two overhead connections [27]. It is noteworthy that there is a small 3_10_ helix (marked as sA, P27-L29) on the long AB loop of ChIFN-α. Additionally, there are two 3_10_ helices (marked as sA’ and sB’, P29-Q33 and E41-K45) on the AB loop of ChIFN-β. The steric position of sA’ is adjacent to sA, located proximal to helix A, but helix sB’ is located in proximity to helix B. It has been proposed that the AB loops of both human IFN-α2a and human IFN-β compose a critical portion of their IFNAR2 binding sites [28], [29]. Whether the differential location of small helices between the two ChIFNs is responsible for distinctive IFNAR2 binding affinity remains to be verified functionally and crystallographically.

### Greater ChIFN-β Expression than ChIFN-α is Induced by Viruses

**Figure 2:**
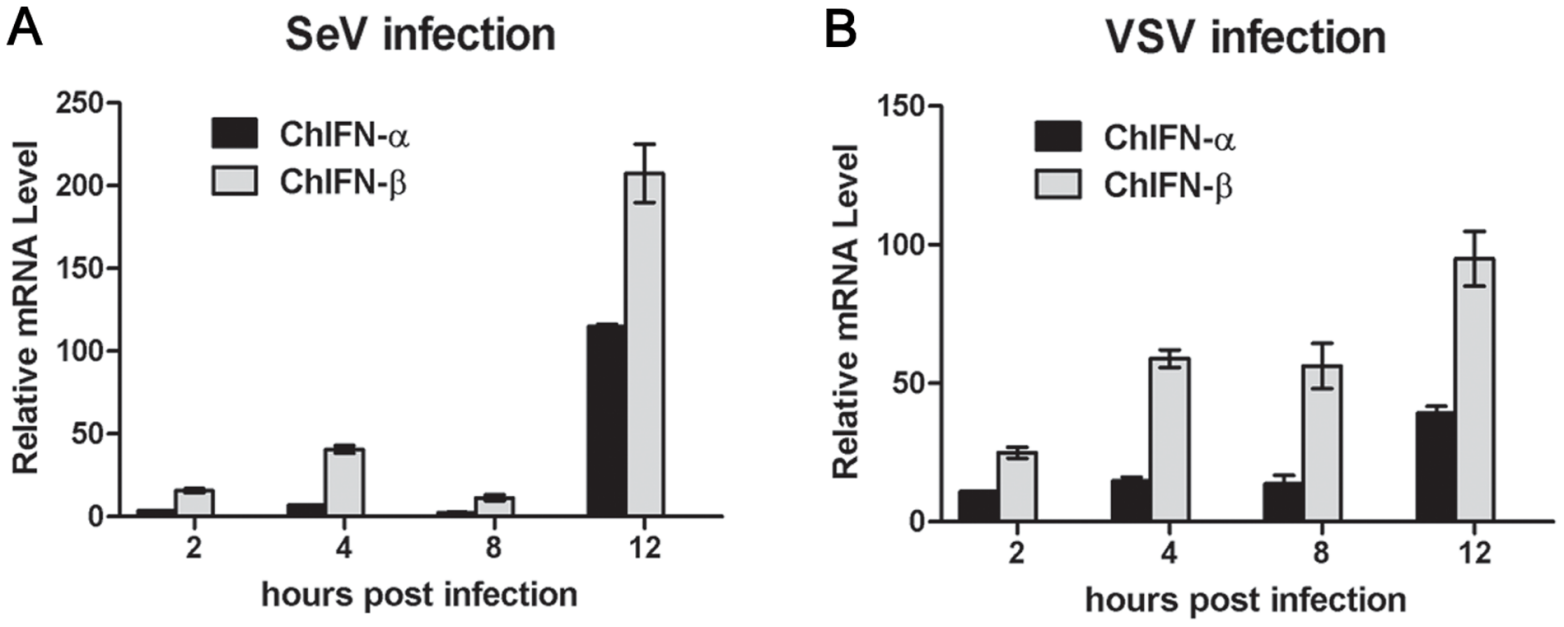
Virus-induced expression of ChIFN-α and ChIFN-β. DF-1 cells were infected with SeV (A) or VSV (B). At the indicated time post infection, cells were harvested for RNA isolation, and virus-induced expression of ChIFN-α or ChIFN-β was assayed by real-time PCR. Data were shown as mean ± SEM (n = 3).

IFN production is one of the most important host responses upon most, if not all, viral infections, with the exclusion of henipavirus infection in fruit bat cell lines that do not induce IFN expression [30]. The negative-sense ssRNA virus Sendai virus (SeV) is considered a strong inducer of type I IFN production [18], and distinct mammalian IFN genes respond differentially to viral induction [31]. In the present study, we determined if both ChIFN-α and ChIFN-β were induced by viral infection in chicken fibroblasts DF-1 cells, and the relative mRNA production of these two ChIFNs was compared. The results demonstrated that expression of both ChIFN-α and ChIFN-β was induced by SeV and VSV infection (Figure 2). In both SeV-infected and VSV-infected DF-1 cells, ChIFN-β expression was induced to a high level with rapid kinetics compared to ChIFN-α. As expected, SeV infection elicited relatively stronger ChIFN production than VSV infection; the peak of ChIFN mRNA induced by SeV infection was approximately 2-fold greater than that induced by VSV infection. In addition to SeV and VSV, influenza virus (H5N1) also induced a more remarkable upregulation of ChIFN-β than ChIFN-α in chicken lung, as reported previously [32]. The differential virus-induced mRNA production of ChIFN-α and ChIFN-β may imply the different involvement of these two ChIFNs in antiviral responses.

### Antiviral Activities of ChIFN-α and -β on DF-1 Cells

**Figure 3:**
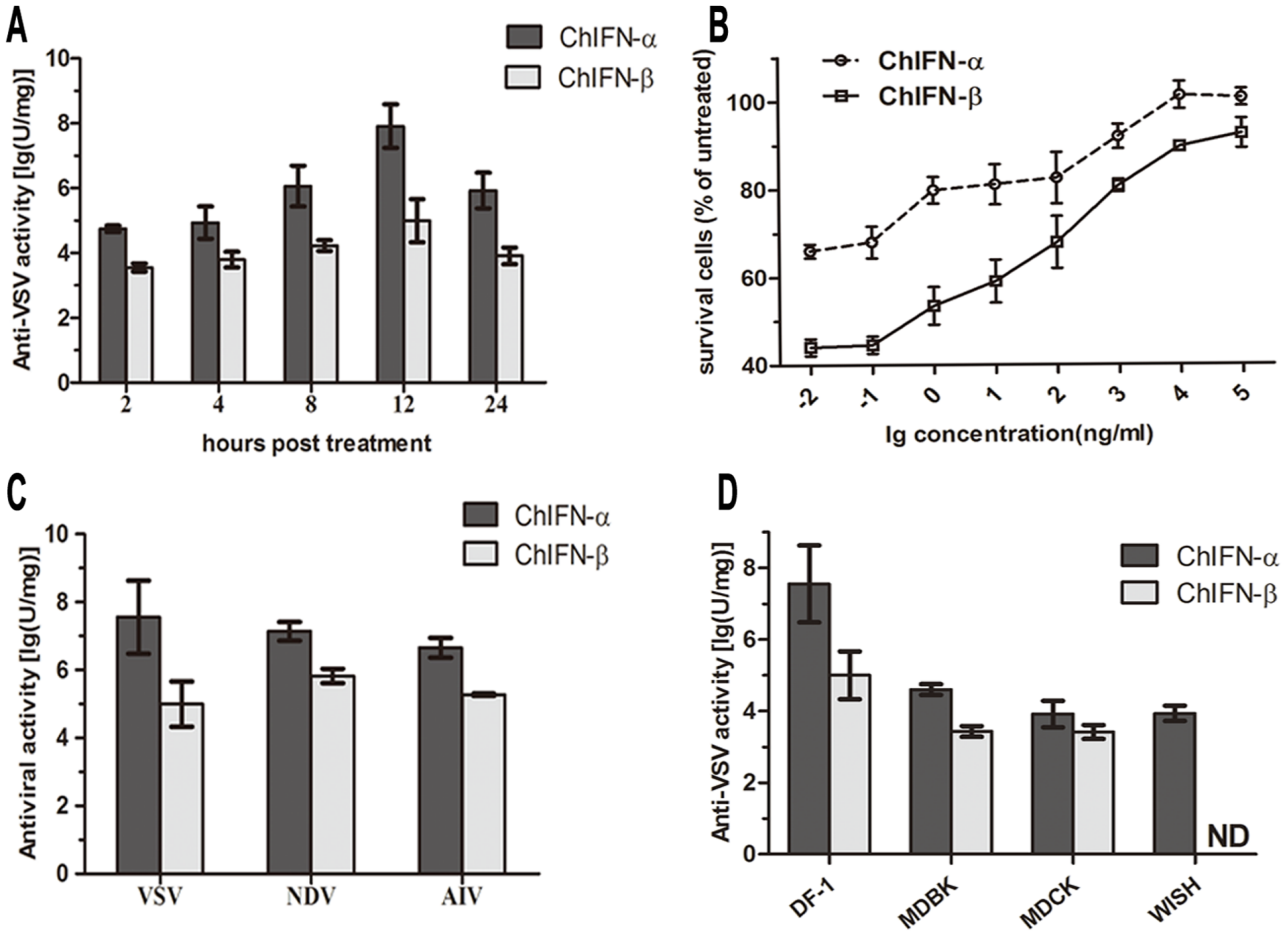
Comparison of the antiviral activities of ChIFN-α and ChIFN-β on DF-1 cells by the CPE inhibition assay. (A) Anti-VSV activities of ChIFNs at different time points. Cells were pretreated with serial four-fold dilutions of ChIFN-α or ChIFN-β for different durations (2, 4, 8, 12, or 24 h) before VSV infection and then stained with crystal violet. (B) Antiviral titration curves of the ChIFNs. DF-1 cells were untreated or treated with 10-fold serial dilutions of ChIFN-α or ChIFN-β for 12 h before VSV infection and then stained with crystal violet. The relative survival percentage was calculated as the ratio of OD values between IFN-treated wells and untreated wells. (C) Antiviral activities of ChIFNs against VSV, NDV and AIV. Cells were treated with serial four-fold dilutions of ChIFN-α or ChIFN-β for 12 h, followed by VSV, NDV or AIV infection, and then stained with crystal violet. (D) Anti-VSV activities of ChIFNs on different cell lines. DF-1, MDBK, MDCK, and WISH cells were treated with serial four-fold dilutions of ChIFN-α or ChIFN-β for 12 h, followed by VSV infection, and staining with crystal violet. The antiviral activities of ChIFN-α and ChIFN-β were represented as the reciprocal of dilutions that led to 50% virus-induced cells lysis. Data were shown as mean ± SD (n = 3). ND: the activity was undetectable.

To compare the antiviral activities of ChIFN-α and ChIFN-β, we first assessed the anti-VSV activities of both proteins at different time points via the cytopathic effect (CPE) inhibition assay on DF-1 cells. The anti-VSV activity of ChIFN-α was ∼100-fold greater than that of ChIFN-β regardless of treatment duration (Figure 3A). It is remarkable that the anti-VSV activities of both ChIFNs displayed a similar temporal pattern, increasing at the beginning of treatment, reaching peak levels at 12 h post treatment (p.t.), and then decreasing. Aside from the antiviral titer of IFN, we also calculated the percentage of survival cells relative to untreated cells to generate antiviral curves. As shown in Figure 3B, the survival percentage of ChIFN-α-treated cells was much higher than ChIFN-β-treated cells, and the advantage was more significant at low concentrations. To verify that the antiviral activity of ChIFN-α is broadly greater than that of ChIFN-β, we further evaluated the activities of ChIFN-α and ChIFN-β against different viruses on DF-1 cells and their anti-VSV activities on different cell lines. Both ChIFN-α and ChIFN-β were biologically active against the selected viruses on DF-1 cells. When assayed in parallel, the antiviral activity of ChIFN-α was greater than that of ChIFN-β. The anti-VSV titer of ChIFN-α reached 10^8 ^U/mg, approximately 1000-fold higher than that of ChIFN-β, a much more notable difference than previously reported [3]. The anti-NDV titer, together with the anti-AIV titer, of ChIFN-α was ∼100-fold greater than that of ChIFN-β (Figure 3C). Furthermore, the anti-VSV activities of ChIFN-α were more powerful than ChIFN-β when assayed on other cell lines (Figure 3D). As expected, ChIFNs exhibited more effective anti-VSV capacity on the homogeneous DF-1 cell line than on the other cell lines, and the anti-VSV capacity of ChIFN-β was undetectable on the human amnion cell line WISH which is considered as a standard cell line for human IFN antiviral assays [33].

### ChIFN-α Induces Higher Expression of ISGs than ChIFN-β

**Figure 4:**
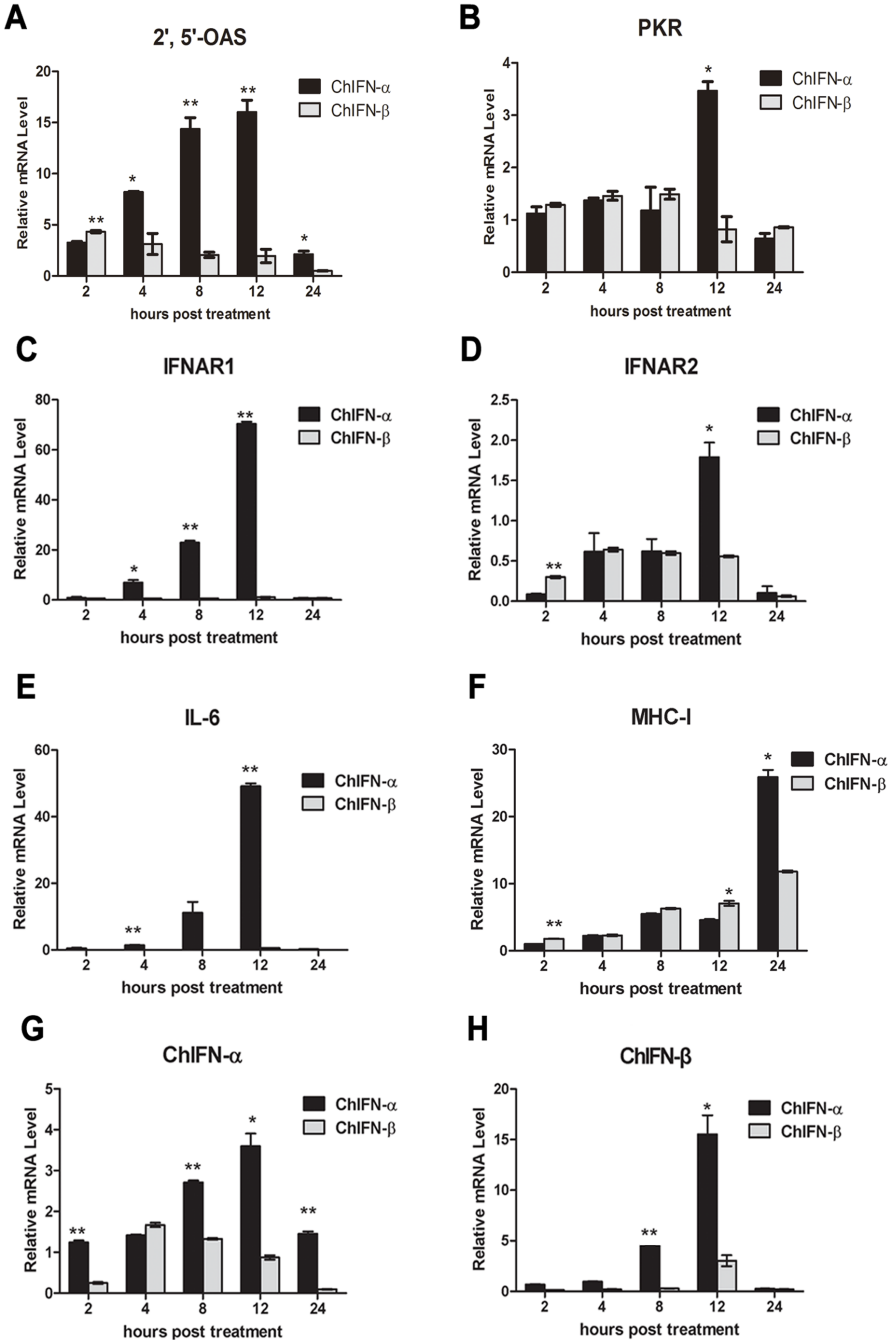
The greater induction potency of ChIFN-α than ChIFN-β on ISGs involved in host defense. After DF-1 cells were incubated with 10 U/ml ChIFN-α or β, the cells were harvested at the indicated times post treatment for RNA extraction and cDNA preparation. The transcriptional levels of 2′,5′-OAS (A), PKR (B), IFNAR1 (C), IFNAR2 (D), IL-6(E), MHC-I (F), IFN-α (G) and IFN-β (H) were assayed by real time PCR. Data were shown as mean ± SEM (n = 3). The Mann-Whitney *U* test was used to compare the differences in relative mRNA levels between ChIFN-α and ChIFN-β treatments at the same time points. A value of *P*<0.05 was considered statistically significant. *P<0.05 and **P<0.01. The asterisks were masked on the top of higher columns.

Differential expression of ISGs accounted for the majority of the different antiviral activities of ChIFN-α and ChIFN-β. In the present study, we concentrated on comparing the induction potency of ChIFN-α and ChIFN-β on 2′,5′-OAS, PKR, IFNAR1, IFNAR2, IL-6 and MHC-I, which are representative ISGs involved in host defense. 2′,5′-OAS and PKR are two important antiviral proteins responsible for the IFN antiviral effect, both activated by dsRNA, and function through degradation of viral mRNA and halting translation, respectively [34], [35]. The mRNA levels of ISGs in respective ChIFN-α- or -β-treated DF-1 cells were relatively quantified by real-time PCR. Following ChIFN-α treatment, 2′,5′-OAS mRNA stably increased during the early portion of the treatment course, reaching a peak of 20-fold greater expression at 12 h p.t. (Figure 4A), which is consistent with the highest antiviral activity at 12 h p.t. in Figure 3A. After ChIFN-β treatment, 2′,5′-OAS, was modestly up-regulated from 2 h p.t. to 8 h p.t., but then the mRNA levels exhibited a decreasing tendency. Comparing the expression of 2′,5′-OAS induced by ChIFN-α and ChIFN-β, we found that the induction effect of ChIFN-β on 2′,5′-OAS was prompt, but not as efficient and durable as ChIFN-α. Concerning ChIFN-induced PKR expression (Figure 4B), with either ChIFN-α or ChIFN-β treatment, the mRNA level was slightly altered with the exception that it was up-regulated approximately 3.5- fold at 12 h post-ChIFN-α treatment.

IFNAR1 and IFNAR2 are essential elements in the type I IFN signaling pathway, transducing extracellular IFN signals to intracellular molecules [36]. In the present study, the transcription of the IFNAR1 gene was extraordinarily activated by ChIFN-α, displaying time-dependent up-regulation from 2. to 12 h p.t. (up to ∼70-fold at 12 h p.t.). However, INFAR1 expression was not up-regulated by ChIFN-β (Figure 4C). The ChIFN-primed IFNAR2 transcription was weak: only 1.79-fold up-regulated at 12 h post-ChIFN-α stimulation and not up-regulated by ChIFN-β (Figure 4D). These results demonstrated that the induction potency of ChIFN-α on IFN receptors was more efficient than that of ChIFN-β, and the induction of IFNAR1 was much more prominent than IFNAR2.

The IL-6 gene was identified as an ISG by microarray results from human dendritic cells and mouse embryonic fibroblasts (MEFs) and is involved in host defense and immune modulation [17], indicating that this inflammation factor contributes to the IFN cellular effect. In this study, the transcription of IL-6, similar to IFNAR1, was sharply and time-dependently enhanced by ChIFN-α treatment but not by ChIFN-β (Figure 4E).

Antigen presentation is a fundamental process in host defense. Expression of MHC class I is induced by IFN in MEFs and mouse lymphocytes [17], [37], and it is also up-regulated by flavivirus and IBDV infection [38], [39], indicating that MHC-I is involved in the antiviral responses. In our study, both ChIFN-α and ChIFN-β induced chicken MHC-I transcription in a time-dependent manner (Figure 4F), but neither stimulated MHC-II expression (data not shown). The induction potency of ChIFN-α on MHC-I was significantly greater than that of ChIFN-β at 24 h p.t., more than 2-fold greater than that achieved by ChIFN-β treatment. The data indicated that ChIFN-α may exert more impact on MHC-I-mediated antigen presentation than ChIFN-β.

Transcription of type I IFNs can be regulated by IFNs themselves in a positive-feedback fashion through IRFs, amplifying the cellular effect of IFNs in an autocrine manner [18]. In this regard, the mRNA levels of the endogenous type I ChIFN genes were also assayed after extracellular ChIFNs treatment. As shown in Figure 4G, endogenous ChIFN-α was transcriptionally up-regulated by extracellular ChIFN-α during the course of the entire assay, whereas, only slightly up-regulated by extracellular ChIFN-β for a short duration (4 to 8 h p.t.). Endogenous ChIFN-β was induced by extracellular ChIFN-α up to ∼15-fold, but only 2-fold up-regulated after 12 h of ChIFN-β treatment (Figure 4H). In brief, ChIFN-α was more prominent and sustainable than ChIFN-β with respect to further amplifying type I IFN expression, and the positive-feedback effect of ChIFNs mainly focused on regulating the transcription of ChIFN-α.

### ChIFN-β is More Potent than ChIFN-α for Up-regulating ISGs Involved in Signaling

**Figure 5:**
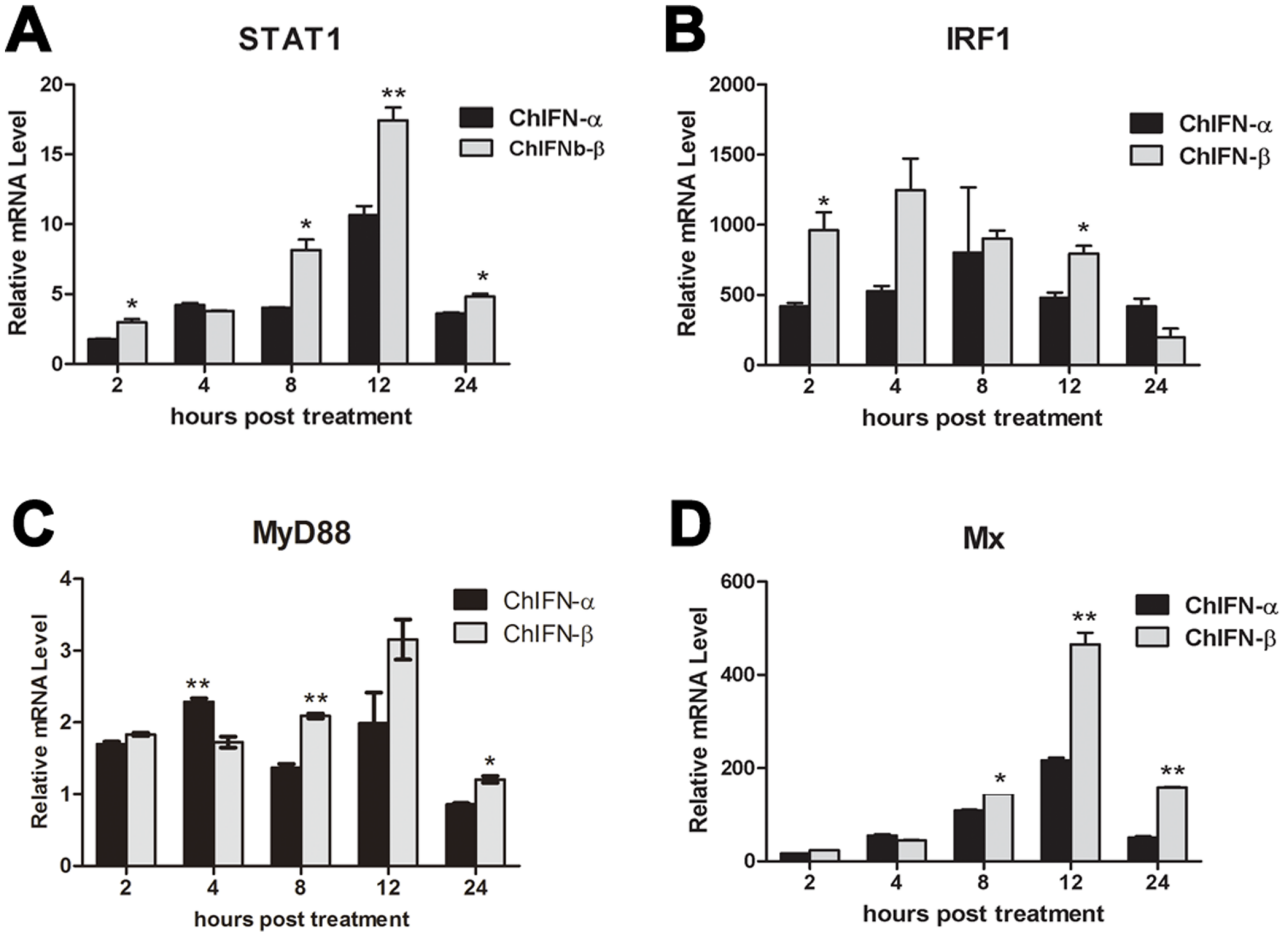
The greater induction potency of ChIFN-β than ChIFN-α on ISGs involved in signaling. After DF-1 cells were incubated with 10 U/ml ChIFN-α or -β, the transcriptional levels of STAT1 (A), IRF1 (B), MyD88 (C) and Mx (D) were assayed at the indicated times post treatment by real time PCR. Data were shown as mean ± SEM (n = 3). The Mann-Whitney *U* test was used to compare the differences in relative mRNA levels between ChIFN-α and ChIFN-β treatments at the same time points. A value of *P*<0.05 was considered statistically significant. *P<0.05 and **P<0.01. The asterisks were masked on the top of higher columns.

Aside from the six ISGs assayed above, many another important ISGs belong to the “host defense” category, the second largest category of ISGs database, such as STAT1, IRF1, MyD88, and Mx. Furthermore, STAT1, IRF1, and MyD88 are also included in the “signaling” category [17]. Mx proteins are IFN-induced GTPases and exhibit antiviral activity against influenza virus [40]. However, chicken Mx is apparently devoid of antiviral activity [41], [42], [43]. Thus, we further compared the induction potency of ChIFN-α and ChIFN-β on these four representative ISGs. As shown in Figure 5, all of the four ISGs were significantly up-regulated by both ChIFN-α and ChIFN-β, though the ChIFN-induced MyD88 signal was relatively weak (Figure 5). ChIFNs induced expression of STAT1 and Mx was similar to that of IFNAR1 and IL-6, peaking at 12 h p.t. However, the expression of IRF1 and MyD88 did not follow the typical time-dependent trend. Comparing the induction potency between these two ChIFNs in Figure 5, we surprisingly found that ChIFN-β was more potent than ChIFN-α. The inverse patterns in Figures 4 and 5 demonstrated that ChIFN-α was not a strictly stronger ISG-inducer than ChIFN-β with regard to all ISGs, though ChIFN-α possessed greater antiviral activity than ChIFN-β.

**Figure 6:**
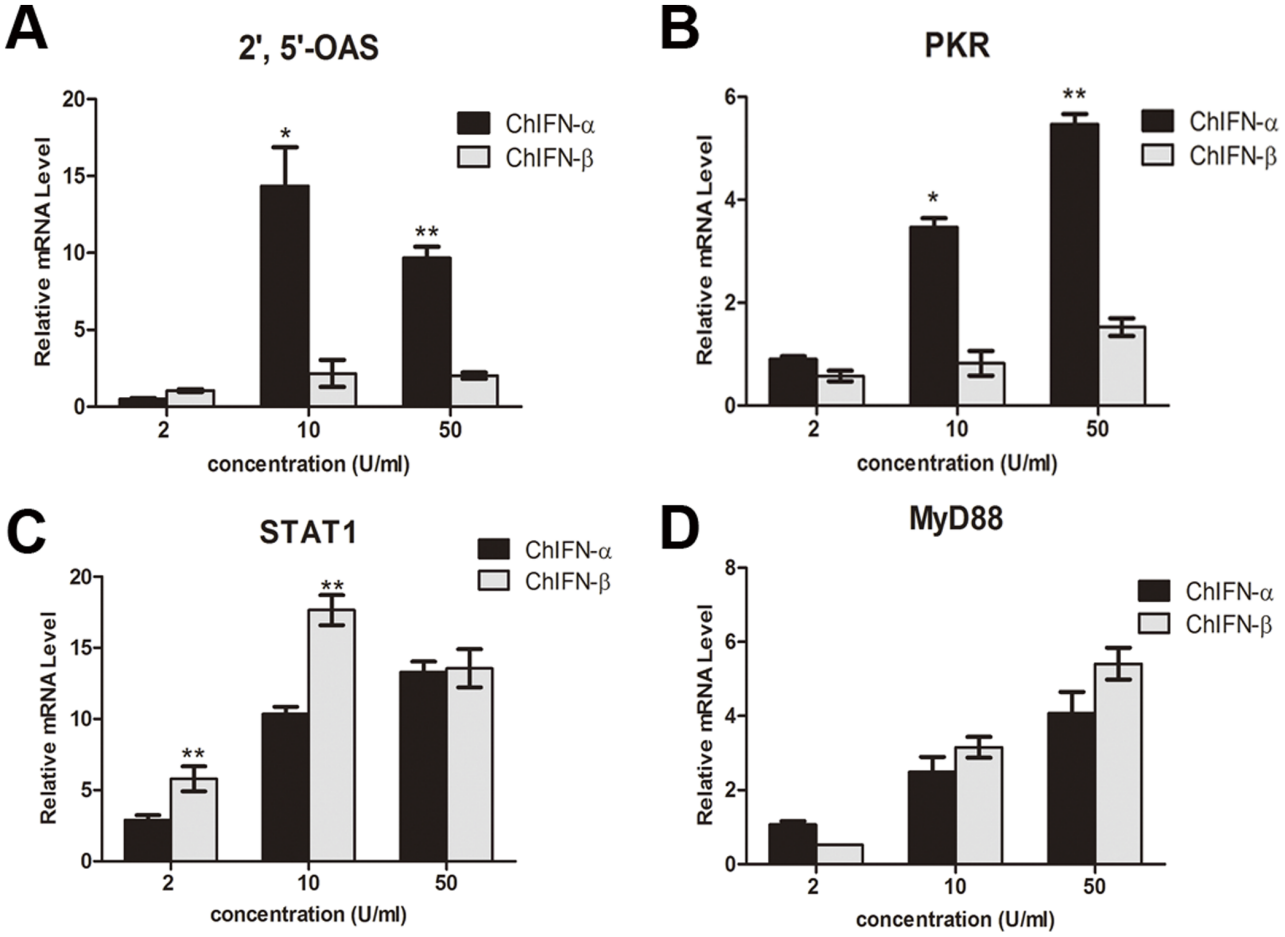
Dose-dependent ISG-inducing potency of ChIFN-α and ChIFN-β. After DF-1 cells were incubated with 2, 10, and 50 U/ml ChIFN-α or ChIFN-β, the cells were harvested at 12 h post treatment for RNA extraction and cDNA preparation. The mRNA levels of 2′,5′-OAS (A), PKR (B), STAT1 (C), and MyD88 (D) were assayed by real time PCR. Data were shown as mean ± SEM (n = 3). The Mann-Whitney *U* test was used to compare the differences in relative mRNA levels between ChIFN-α and ChIFN-β treatments at the same time points. A value of *P*<0.05 was considered statistically significant. *P<0.05 and **P<0.01. The asterisks were masked on the top of higher columns.

To ascertain how ChIFN-α and ChIFN-β, respectively, induced the expression of ISGs, we further determined the mRNA levels of 2′,5′-OAS, PKR, STAT1, and MyD88 induced by multiple doses of the ChIFNs at the same time point. As shown in Figure 6, ISG expression induced by both ChIFN-α and ChIFN-β exhibited a dose-dependent trend, except expression of 2′,5′-OAS and STAT1 induced by ChIFN-β. The minimal up-regulation by the high dose of ChIFN-β was likely due to the toxicity of IFN at high concentration. In accordance with Figures 4 and 5, ChIFN-α was more potent than ChIFN-β for the up-regulation of 2′,5′-OAS and PKR, but less potent than ChIFN-β for STAT1 and MyD88.

## Discussion

Mounting evidence demonstrates that different members of the type I IFN family function distinctively through the same receptors, such as human and feline type I IFNs [11], [44]. Chicken type I IFNs followed this pattern in the present study, exhibiting significantly different antiviral activities despite possessing high conservation in terms of sequence and predicted structure. We systematically compared the antiviral activities of recombinant ChIFN-α and ChIFN-β for the first time. To elucidate how ChIFN-α and ChIFN-β functioned differentially, we quantified the ISGs at the transcriptional level. Products of the ISGs provide a molecular marker for evaluating the antiviral status of cells [45], [46]. In this study, the chosen ISGs encoded critical proteins that function in host defense. As reported [47], the IFN-inducible antiviral proteins 2′,5′-OAS and PKR are up-regulated as part of the host protective response when chickens were infected with AIV H9N2. In human hepatoma culture HepG2.2.15 cells, PKR facilitates HBx siRNA-mediated inhibition of HBV replication [48]. In H5N1-infected chickens, transcription of PKR is activated [49]. These data indicate that 2′,5′-OAS and PKR play important roles in inhibiting virus replication in chickens and other mammals. In this study, the mRNA levels of 2′,5′-OAS and PKR indicated that the antiviral status induced by ChIFN-α was stronger than that of ChIFN-β, which was in accordance with the higher antiviral titer of ChIFN-α compared to ChIFN-β.

The IFNAR1 and IFNAR2 genes have been incorporated into the ISGs database [17]. IFNAR1 and IFNAR2 confer antiviral activities against VSV and are up-regulated after IBDV infection [39], [50]. Furthermore, the replication of influenza virus increases in MEFs lacking type I IFN receptors [51], which also implies that IFNAR1 and IFNAR2 take part in the antiviral response. In our study, IFNARs were divergently regulated upon ChIFN-α and ChIFN-β treatment, which may be another indicator of the differential antiviral activities displayed by ChIFN-α and ChIFN-β. Remarkably, the binding affinity between IFNAR2 and IFN is high, with a dissociation constant in nanomolar range, whereas the affinity between IFNAR1 and IFN is in the micromolar range [52], hypothetically because the demand for IFNAR1 in stable IFN-receptor complexes is greater than that for IFNAR2. Recent research shows that the fold change of IFNAR1 up-regulation is greater than IFNAR2 during development in chicken lung and spleen [53], supporting the above hypothesis. As shown in Figure 4C and D, the induction of IFNAR1 by ChIFNs is more prominent than that of IFNAR2, which is also consistent.

Influenza virus infection can induce IL-6 expression in chickens, macaques and ferrets [49], [54], [55], which implies the importance of IL-6 in antiviral defense. In the present study, ChIFN-α but not ChIFN-β significantly up-regulated IL-6, which is an ISG family member. More significantly, ChIFN-α did not induce expression of IL-8 (data not shown), another member in the ISG database derived from human and murine cell lines. The lack of IL-8 induction indicates that the ISGs database results derived from microarray experiments of human and murine cell lines do not completely match the real time results from chicken cells whose cadre of ISGs remain to be determined.

Moreover, the feedback effects of chicken type I IFNs were assayed in this study. Recombinant ChIFN-α treatment significantly up-regulated endogenous ChIFN-α and ChIFN-β, whereas, the positive-feedback effect of ChIFN-β was much less. This is the first comparison of the feedback effects of ChIFN-α and ChIFN-β, which may provides important information for elucidating the differential bioactivities of ChIFN-α and ChIFN-β.

Another four representative ISGs (STAT1, IRF1, MyD88, and Mx) were assayed to confirm the differential potency of ChIFN-α and ChIFN-β. STAT1 is a critical component in the type I IFN signaling pathway, which transduces cytoplasmic signals to the nucleus and activates gene expression through IFN-stimulated response elements (ISREs) [16]. As mentioned above, IRFs are indispensable for IFN production. It has been reported that IRF1 can interact with MyD88, which is a pivotal adaptor for all Toll-like receptors (TLRs) except TLR3 [56]. Mx is an IFN-inducible GTPase and an antiviral protein in mice, though additional research proves that chicken Mx does not inhibit AIV replication [41], [42]. We were surprised to find that the induction potency of ChIFN-α on these four ISGs involved in signaling was less than that of ChIFN-β, a contrary result to the potency shown in Figure 4. The opposite results in Figures 4 and 5 imply the existence of diverse effect of ChIFNs on different ISGs, and we further speculate that ChIFN-β may be mainly committed to signaling and immune modulation rather than directly to the antiviral response, which remains to be verified.

It is notable that chicken IFN induction potency in the present study did not absolutely conform to human IFN induction potency on several ISGs obtained by microarray, demonstrating that human IFN-β treatment results in a higher fold change in PKR, 2′,5′-OAS, and MHC-I mRNAs than IFN-α treatment [11], [45]. Even within the human type I IFN family, there are controversial results in different cell lines: IFN-β is more potent than IFN-α in human melanoma cells, but equivalent effect from IFN-α and IFN-β stimulation have been observed in human dermal microvascular endothelial cells [11], [57]. Furthermore, concerning to the same ISG, the induction potency of ChIFN-α and ChIFN-β varied between different cell types. Chicken type I IFN induces TLR3 upregulation in DF-1 cells but not in HD11 cells [58]. Phylogenetically, chicken type I IFNs form an outgroup of mammalian type I IFNs, i.e., they are evolutionarily distant from human IFNs, and ChIFN-β is a separate branch of the ChIFN-α family [59]. Taking evolutionary aspects into consideration, it is reasonable to accept the opposite results between chicken and human type I IFNs, as well as the different activities between ChIFN-α and ChIFN-β.

## Materials and Methods

### Virus Stocks and Cells

AIV subtype H9N2, isolate A/Chicken/LiaoNing/1/00, was a gift of Professor Jinhua Liu (College of Animal Medicine, China Agricultural University, Beijing, China). VSV and the NDV LaSota strain were purchased from Harbin Veterinary Research Institute, Chinese Academy of Agricultural Sciences. AIV and NDV were propagated in 10-day-old SPF chicken embryonated eggs; VSV was propagated in Madin-Darby bovine kidney (MDBK) cells (ATCC CCL-22). The allantoic fluids and cell culture medium were harvested and stored at −70°C and were used for testing 50% tissue culture infective doses (TCID_50_).

The chicken fibroblast cell line DF-1 (ATCC CRL-12203), MDBK cell line, the Madin-Darby canine kidney (MDCK) cell line (ATCC CCL-34) and the human amnion WISH cell line (ATCC CCL-25)were cultured in Dulbecco’s modified Eagle’s medium (DMEM, GIBICO) supplemented with 10% heat-inactivated fetal bovine serum (FBS, GIBICO).

### Preparation of ChIFN-α and ChIFN-β

The ChIFN-α gene lacking its signal sequence was amplified by PCR from the pBV220/ChIFN-α plasmid [24]. The forward primer was 5′-GGAATTCCATATGTGCAACCACCTTCGCC-3′, and the reverse primer was 5′-CCGGAATTCCTAAGTGCGCGTGTTGC-3′. The ChIFN-β gene lacking its signal sequence was amplified by PCR from the cDNA of VSV-infected DF-1 cells. The forward primer was 5′- GGAATTCCATATGTGCAACCATCTTCGTC -3′, and the reverse primer was 5′- CCGGAATTCTCACTGGGTGTTGAGAC -3′. cDNA from VSV-infected DF-1 cells was synthesized as described previously [24]. Briefly, total RNA of VSV-infected DF-1 cells was first isolated using TRIzol reagent (Invitrogen) and then immediately converted into cDNA by AMV reverse transcriptase (Promega).

Both ChIFN-α and ChIFN-β PCR products were cloned into the *Nde*I/*Eco*RI sites of the pET28b vector (Novagen, German) to generate the recombinant plasmids pET28b/ChIFN-α and pET28b/ChIFN-β. These sequences of two plasmids were confirmed by DNA sequencing, transformed into *E. coli* strain BL21 (DE3), and protein production was induced with 1 mM IPTG for 4 h at 37°C. Recombinant ChIFN-α and ChIFN-β were purified as described previously [24] with minor modifications. Briefly, the cells were harvested by centrifugation and resuspended in phosphate buffered saline (PBS), followed by sonication. Inclusion bodies were isolated by centrifugation and washed three times with PBST (PBS supplemented with 1% Triton X-100), 2 M urea, and 1 M NaCl by turns, then dissolved in 6 M guanidine hydrochloride. The insoluble portion was removed after centrifugation, and the soluble protein was refolded by a 20-fold dilution in refolding buffer: 50 mM Tris, (pH 8.0), 0.4 M L-Arg, 2 mM EDTA, 0.5 mM glutathione (oxidized), 5 mM glutathione (deoxidized), and 10% glycerol. After 48 h of incubation at 4°C, the solution was concentrated and purified by gel filtration. The purified recombinant proteins were detected by SDS-PAGE and western blot analysis.

### Antiviral Activity Assay of ChIFN-α and ChIFN-β

The antiviral titers of ChIFN-α and ChIFN-β were measured by the CPE inhibition assay in systems comprised of two groups: VSV/DF-1, NDV/DF-1, AIV/DF-1 and VSV/DF-1, VSV/MDBK, VSV/MDCK, VSV/WISH. In brief, cells were seeded in 96-well microplates at a concentration of 10^4^ cells per well and cultured at 37°C in humid air with 5% CO_2_ for 10 h. Monolayers of DF-1 cells were stimulated with 100 µl of four-fold serial dilutions of ChIFN-α or ChIFN-β. Recombinant ChIFN-α or ChIFN-β were treated with a ToxinEraser™ Endotoxin Removal Kit (GenScript) to remove LPS (according to manufacturer’s directions) before dilution. After various times (2, 4, 8, 12 and 24 h) of culture, the cells were challenged with 100 TCID_50_ viruses (VSV, NDV or AIV) per well and cultured until the CPE of virus-infected cells without ChIFN treatment appeared. Cultures were stained with crystal violet. The ChIFN titers (U/mg) are expressed as the reciprocal of the dilutions that led to 50% virus-induced cells lysis by the Reed-Muench method [2].

### Quantified Real-time PCR of Virus-induced Chicken Type I IFNs and ChIFNs-induced ISGs in DF-1 Cells

To detect virus-induced expression of ChIFN-α and ChIFN-β, DF-1 cells were infected with VSV or SeV at an m.o.i. of 0.01 or 10, respectively. Cell samples were collected at 2, 4, 8 and 12 h after infection for RNA isolation. The RNA samples were treated with DNase I (TaKaRa) at 37°C for 30 min. Total RNA was reverse transcribed to cDNA using AMV reverse transcriptase (Promega) and 10 pmol of oligo-dT primer according to the manufacturer’s direction. The exonuclease activity of AMV reverse transcriptase was then heat-inactivated at 95°C for 5 min. Negative controls (amplifications in the absence of RNA or primers) were included in parallel to confirm the absence of contamination by template nucleic acids and the efficiency of RT inactivation. cDNA was aliquot and stored at −80°C. cDNA from uninfected cells was used as a calibrator (set as 1) to evaluate the mRNA levels of virus-induced ChIFN-α and ChIFN-β. The resulting mRNA levels were normalized to the housekeeping gene β-actin.

To investigate the mRNA levels of chosen ISGs at different time points after ChIFN-treatment, DF-1 cells were treated with 10 U/ml ChIFN-α or ChIFN-β for the indicated durations. The antiviral units of ChIFN-α and ChIFN-β were determined according to the anti-VSV activity at 12 h post-IFN treatment. Cell samples were collected at 2, 4, 8, 12 and 24 h post-ChIFNs treatment for RNA extraction, cDNA preparation, and real-time PCR. Untreated DF-1 cells were collected at 0 h and used as a calibrator to evaluate the mRNA levels of chosen ISGs; β-actin was used as an internal control.

Specific primers for β-actin, PKR, 2′, 5′-OAS, IFNAR1, IFNAR2, IL-6, IL-8, ChIFN-α, ChIFN-β, IRF1, MyD88, STAT1, Mx, and MHC-I are described in precious studies [24], [39], [49], [60], [61]. RNA isolation and real-time PCR were processed according to previous studies [62], [63].

### Structure Modeling of ChIFN-α and ChIFN-β

The three-dimensional structures of ChIFN-α and ChIFN-β were modeled using a protein structure homology-modeling server (available at http://swissmodel.expasy.org/) in automated mode [64]. Human IFN-α2 (PDB-ID: 2KZ1, chain A) and IFN-β (PDB-ID: 1AU1) were used as templates for ChIFN-α and ChIFN-β modeling, respectively. The modeled structures were viewed and analyzed using PyMOL.

### Statistical Analysis

All statistical analyses were performed using SPSS version 13.0 (SPSS Inc., Chicago, IL). Comparisons between different treatments were performed using the Mann-Whitney *U* test. Two-sided *P*<0.05 was considered to be significant for all tests.
